# Elucidation of *Callistemon lanceolatus*-derived natural compounds in STAT 3 pathway against human cancer cells: *in silico* and *in vitro* studies

**DOI:** 10.3389/fphar.2025.1507002

**Published:** 2025-02-21

**Authors:** Mubashir Zafar, Sadaf Anwar, Malik Asif Hussain, Naveed Iqbal, Abrar Ali, Sadaf, Mohammad Zeeshan Najm, Mohd Adnan Kausar

**Affiliations:** ^1^ Department of Family and Community Medicine, College of Medicine, University of Ha’il, Hail, Saudi Arabia; ^2^ Department of Biochemistry, College of Medicine, University of Ha’il, Hail, Saudi Arabia; ^3^ Department of Pathology, College of Medicine, University of Ha’il, Hail, Saudi Arabia; ^4^ Department of Obstetrics and Gynecology, College of Medicine, University of Ha’il, Hail, Saudi Arabia; ^5^ Department of Ophthalmology, College of Medicine, University of Ha’il, Hail, Saudi Arabia; ^6^ Department of Biotechnology, Jamia Millia Islamia, New Delhi, India; ^7^ School of Biosciences, Apeejay Stya University, Gurugram, Haryana, India

**Keywords:** STAT3, cancer, molecular docking, MD simulation, apoptosis

## Abstract

**Introduction:**

Many human tumours have hyperactive signal transducer and activator of transcription 3 (STAT3, positioning it as a prime target for natural compounds with anticancer properties. This study investigated three small-molecule STAT3 inhibitors derived from *Callistemon lanceolatus*: cyanidin-3,5-diglucoside, kaempferol-3-o-β-d-galactopyranoside, and quercetin-3-o-(2″-o-galloyl)-β-d-galactopyranoside.

**Material and methods:**

The compounds were explored through virtual screening and molecular dynamics (MD) simulations to understand the intracellular processing of activated STAT3. The biological effects of these STAT3 inhibitors on human cancer cells were assessed via simulation. Further, *in-vitro* studies were performed to exhibit the anti-cancer role of compounds on cancer cell lines.

**Results and discussion:**

It is revealed through results that active components in these compounds inhibited cancer cell migration and invasion and suppressed the proliferation of noncancer cells. Moreover, these natural compounds from *C. lanceolatus* downregulated the expression of STAT3 downstream target proteins, indicating their potential as therapeutic agents against cancer. Thus, cyanidin-3,5-diglucoside, kaempferol-3-o-β-d-galactopyranoside, and quercetin-3-o-(2″-o-galloyl)-β-d-galactopyranoside from *C. lanceolatus* are promising candidates for cancer treatment.

## 1 Introduction

Cancer remains one of the most severe diseases globally, claiming more lives than any other disease (Chhikara and Parang, 2023). Cancer is increasingly recognised to arise from somatic mutations and inherited genetic alterations. Environmental factors and lifestyle choices, such as exposure to pollutants, radiation, alcohol, tobacco use, chronic infections, obesity, and a high-calorie diet, contribute to cancer development by affecting cellular pathways involved in growth, differentiation, and apoptotic protein expression (Biswas et al., 2022). Advances in understanding molecular mechanisms through which these carcinogens operate are unfolding. Many of these risk factors lead to chronic inflammation, a critical element in the process of carcinogenesis. A study indicated that chronic inflammation is associated with cancer because crucial risk factors can activate proinflammatory transcription factors, such as NF-κB and STAT3 (Perez et al., 2020). Given their pivotal roles in carcinogenesis, these transcription factors are being considered as potential targets in the prevention and treatment of cancer (Darnell, 2005).

The STAT3 pathway has been identified as a potential target for many cancers, including cervical cancer, lymphomas, hepatocellular carcinoma, multiple myeloma tumours, and leukemia, (Bowman et al., 2000). Although STAT3 inhibits apoptosis in normal cells, it can stimulate angiogenesis and cell proliferation in cancer cells (Chaffer and Weinberg, 2011). Many benign and malignant tumours in humans have constitutive STAT3 activation due to aberrant growth factor, cytokine receptor, and Janus kinases (JAK) regulation (Fletcher et al., 2008). Multiple cancer cell lines undergo apoptosis, and cell growth stops when STAT3 signalling is inhibited; this strategy discourages tumour regression and kills cancer cells while having no impact on normal cells (Fletcher et al., 2008; Debnath et al., 2012). Thus, STAT3 might be a promising target for cancer treatments.

The current research identified natural compounds that can disrupt STAT3 activity by affecting its translocation, dimerisation, and DNA binding. The literature review indicated that several natural compounds affect the STAT3 signalling pathway, including quercetin, curcumin, betulinic acid, withaferin A, and ursolic acid (Bose et al., 2020).

*Callistemon lanceolatus*, often known as Crimson Bottlebrush, is a well-known natural product with a wide range of therapeutic applications. It is a member of the Myrtaceae family (Mahmoud et al., 2002). This study examined the anticancer effect of the compounds of *C. lanceolatus*. The primary components of *C. lanceolatus* are flavonoids, terpenoids, and phenolic compounds; the exact mechanism through which these substances inhibit STAT3 to regulate angiogenesis remains unknown. These chemicals exhibited antiangiogeneic properties in various plants, including *Podophyllum* species, *Catharanthus roseus*, *Camptotheca acuminate*, and *Taxus brevifolia* (Pu et al., 2020; Sarli et al., 2020; Kumar et al., 2022). Thus, this research included *in vitro* and *in silico* studies to explore the effect of *C. lanceolatus* components on STAT3 inhibition in cancer.

## 2 Materials and methods

### 2.1 *In silico* studies

#### 2.1.1 Potential therapeutic target identification

A total of 38 natural compounds from *C. lanceolatus* were identified from scholarly articles, and the PharmMapper server was used to accurately determine their pharmacological targets (Liu et al., 2010). This user-friendly online platform employs reverse pharmacophore mapping to predict potential targets for small molecules derived from plants. The server is supported by over 7000 target pharmacophore models from databases such as Target Bank, the Potential Drug Target Database, and DrugBank (Liu et al., 2010). To identify the most suitable pharmacophore model for the molecule, a method combining triangle hashing and a genetic algorithm was employed. Researchers used this tool to explore possible interactions between *C. lanceolatus*-derived compounds and the STAT3 transcription factor. The fit score was calculated after the molecular structures in MOL format were submitted to PharmMapper. The search was restricted to human targets using the target parameter, whereas other settings remained at their default values.

#### 2.1.2 Molecular docking

The Protein Data Bank provided the X-ray crystal structure of STAT 3 (PDB ID: 1BG1) (Matsuno et al., 2010). The STAT 3 (PDB ID: 1BG1) was fine-tuned with the help of Auto Dock Tools (ADT) version 1.5.6, which is available from the Scripps Research Institute. Chembio Draw ultra was used to draw the 2D and 3D structures of natural substances and known STAT3 inhibitors, such as sanguinarine and plumbagin (Debnath et al., 2012). Additional computations and file preparations were performed in compliance with published procedures (Morris et al., 1998; Ahmad et al., 2017; Ansari et al., 2018). Following the preparation of the coordinate files for STAT3 and the corresponding compound, the AutoDock 4.2 software was used to dock it (Morris et al., 1998). A grid with a point spacing of 0.375 Å was set up with the auto-grid dimensions of 96 × 96 × 96 Å along the XYZ axis. By default, the Auto-Grid module uses the Lamerckian genetic algorithm (LGA) and empirical force fields to forecast the bound conformation, whereas the RNG is configured with the specified values. Using electrostatic interactions, van der Waals, and hydrogen bonding, the binding energy was estimated. Finally, the docked STAT three complexes were fine-tuned, validated, and investigated with the use of the “Protein-Ligand Interactions” modules in Discover Studio 4.0 and pymol (Studio, 2013).

#### 2.1.3 Molecular dynamics simulations

Molecular Dynamics (MD) simulations were conducted on wild-type STAT3 and its complexes with compounds cyanidin-3,5-diglucoside, kaempferol-3-o-β-d-galactopyranoside, and quercetin-3-o-(2″-o-galloyl)-β-d-galactopyranoside using GROMACS Version 5.1.2 on an HP Proliant DL580 G7 workstation with a GTX 1070 graphics card. The STAT3 topology was generated using the GROMOS96 53A6 force field (Van Der Spoel et al., 2005). Because the compounds and other therapeutic molecules lacked appropriate force field parameters in GROMACS, topology-coordinate files were created using the PRODRG server for the compounds cyanidin-3,5-diglucoside, kaempferol-3-o-β-d-galactopyranoside, and quercetin-3-o-(2″-o-galloyl)-β-d-galactopyranoside. The docked complexes were immersed in a cubic SPC/E water box, requiring the addition of five Cl-counterions to neutralise the system. Each simulation used a water environment with a 2fs timestep. Energy minimisation was achieved using steepest descent and conjugate gradient algorithms, with a convergence criterion of less than 0.005 kcal/mol. The structures were subjected to MD simulation constraints to improve reliability. Following the equilibration phase, each simulation was independently conducted under constant volume (NVT), constant temperature (300 K), and constant pressure (NPT) conditions for 100 ps. The V-rescale thermostat was set at 300 K, and the C-rescale barostat at 1 bar to maintain binding coordinates. Finally, the MD simulations were stabilised at 100 ns per run, totalling 400 ns for four separate simulations. Each trajectory underwent analysis with xmgrace, focusing on metrics such as the radius of gyration (Rg), root mean square fluctuations (RMSF), and root mean square deviation (RMSD).

#### 2.1.4 *In silico* pharmacokinetics analysis

##### 2.1.4.1 ADME properties study

In accordance with a published methodology, ADME values for the last lead chemical screening were generated using Discovery Studio 3.5 (Accelrys San Diego, United States) (Ahmad et al., 2017).

##### 2.1.4.2 QSTR analysis

Studies on quantitative structure toxicity relationships (QSTRs) were examined using the TOPKAT feature included in the DS 3.5 program (Ahmad et al., 2017). To optimise the therapeutic ratio of lead compounds for future research and to identify any possible safety problems, the TOPKAT predictions are useful. In addition to establishing a dosage range for *in vitro* experiments, the values are useful for evaluating contaminants, intermediates, and metabolites.

##### 2.1.4.3 Biological activity spectrum (BAS)

Predictions of biological activity were made using the biological activity spectrum (BAS) method, following a published protocol.

### 2.2 *In vitro* studies

#### 2.2.1 Chemicals

The chemicals quercetin-3-o-(2″-o-galloyl)-β-d-galactopyranoside and cyanidin-3,5-diglucoside were sourced from Medkoo Biosciences and Sigma-Aldrich, respectively. Stock solutions were prepared in DMSO (Merck & Co., India). Primary antibodies for Stat3 (Cat# sc482) and β-actin (Cat # 3700), along with mouse/rabbit secondary antibodies, were purchased from Santa Cruz Biotechnology (United States). HEK293, MCF7, and HeLa cell lines were obtained from the National Centre for Cell Sciences (NCCS) in Pune, India. Dulbecco’s Modified Eagle’s Medium and fetal bovine serum were obtained from Gibco-life technologies, and Trizol and the cDNA synthesis kit were purchased from Bio-Rad (United States).

#### 2.2.2 Cell proliferation assay

The MTT assay was used to assess the effects of the lead compounds on cell viability and proliferation, following established protocols (Mosmann, 1983; Ansari et al., 2016). Briefly, 5,000 to 7,000 exponentially growing HeLa, MCF7, and HEK293 cells were seeded per well in a 96-well plate. The next day, these cells were treated with varying concentrations of the lead compounds (2.5–80 μM for cancer cell lines and 5–200 μM for HEK293 cells). Coumarin was used as a positive control in the cytotoxicity and activity assays. At the end of the incubation period, 20 μL of MTT solution (5 mg/mL in PBS) was added to each well, followed by incubation at 37°C in a humidified chamber for 4 h. The supernatant was then removed, and 100 μL of DMSO was added to dissolve the formed formazan crystals. Absorbance at 570 nm was measured using an ELISA reader (BioRad) to determine cell viability. IC50 values were calculated for each compound using Excel’s best-fit regression curve method. The selectivity index (SI) was determined following the methodology outlined by Fang et al. (2006). All experiments were conducted in triplicate.

#### 2.2.3 Fluorescence microscopic analysis of cell death

Fluorescence microscopy was used to examine the nuclear morphology of HeLa cells post-staining with 4′,6-diamidino-2-phenylindole (DAPI), adhering to the standard methods previously described (Ramar et al., 2012). Briefly, HeLa cells were treated for 48 h with 5 μM and 15 µM concentrations of the compounds in fresh media (control). Subsequently, the cells were washed with PBS (pH 7.4), fixed in 70% ethanol, and then resuspended in 50 µL of DAPI solution (1 μg/mL DAPI in distilled water). The cells were covered with aluminum foil and incubated for 20 min at 37°C. Observation was conducted using a Nikon Eclipse E600 fluorescence microscope.

#### 2.2.4 Cell migration assay

The wound healing assay was performed to evaluate the migratory behavior of HeLa cells following the method described by Singh and Bast (2015). HeLa cells were seeded in a six-well plate and allowed to reach confluence overnight. After 24 h, the medium was replaced with fresh medium containing 0.5% FBS, and the cells were treated with varying concentrations of the compounds (5 μM–15 µM). A scratch was made in the cell monolayer by using a 200-µL pipette tip, followed by incubation at 37°C with 95% air and 5% CO2. After 48 h, to remove any detached cells, the wells were washed twice with DMEM. Images were captured post 48 h, with scale bars added using ImageJ software, and the wound closure rate was quantified.

#### 2.2.5 Total RNA isolation and quantitative RTPCR assay

A six-well plate was used to seed 1 × 10^5^ cells per well, which were then cultured in DMEM supplemented with 10% FBS in a CO_2_ incubator. Prior to the treatment, the cells underwent 6 h of starvation. Afterward, the culture medium was replaced with fresh medium containing 10% FBS, and the cells were treated with compound concentrations ranging from 5 to 15 µM for 48 h. Following the incubation, the cells were harvested and washed three times with PBS. Total RNA isolation was performed using the TRIZOL (Invitrogen) method, involving the collection and triple washing of approximately 1 × 10^6^ cells. The cells were then lysed with 1 mL of TRIZOL reagent, followed by a 5-min incubation. After adding 200 µL of chloroform and mixing by inversion, the mixture rested at room temperature for 5 min before being centrifuged at 13,000 × *g* for 15 min. The aqueous phase was carefully transferred to a new tube, mixed with an equal volume of isopropanol, and centrifuged at 13,000 × *g* for 10 min at 4°C. The resulting pellet was washed with 1 mL of 70% ethanol and centrifuged at 7,500 g for 5 min at 4°C. Once air-dried, the pellet was resuspended in 50 µL of DEPC-treated water. The isolated RNA was treated with DNase (Roche Applied Sciences) to remove any potential genomic DNA contamination. The concentration and purity of the RNA were assessed spectrophotometrically by using the A260/A280 absorbance ratio. The integrity of RNA was verified through agarose gel electrophoresis in MOPS buffer. For cDNA synthesis, 150 ng of RNA was used with the cDNA synthesis kit (BIO-RAD) following the manufacturer’s instructions. RT-PCR was performed using the assay-on-demand method, with primers listed in Table 1 provided by Applied Biosystems. RT-PCR products were verified through 2% agarose gel electrophoresis, and relative mRNA levels were normalised against β-actin.

**TABLE 1:** List of Primer sequences.

Name of gene	Forward primer (5′ to 3′)	Reverse primer (5′ to 3′)
STAT3	GGA​CAT​CAG​CGG​TAA​GAC​CC	CCT​GGG​TCA​GCT​TCA​GGA​TG
p53	TGT​GAC​TTG​CAC​GTA​CTC​CC	ACC​ATC​GCT​ATC​TGA​GCA​GC
β-actin	TCA​GAA​GAA​CTC​CTA​TGT​GG	TCT​CTT​TGA​TGT​CAC​GCA​CG

#### 2.2.6 Cell cycle analysis

Propidium iodide (PI) staining was performed to analyse cell cycle dynamics as previously described [34,35]. HeLa cells were treated with various concentrations of lead compounds (5–15 µM) for approximately 24 h, whereas control cells were incubated with only the complete media. The cells were then trypsinised, washed with cold PBS, and centrifuged for 4 min. Cells were fixed by gently adding pre-chilled 70% ethanol. After overnight incubation at 4°C, the cells were resuspended in 50 μL of PBS and treated with 200 μg/mL RNase A, followed by a 37°C incubation for 50 min. Subsequently, 2 μg/mL of PI was added and incubated for 15 min. Flow cytometry analysis was conducted on each sample by using the Becton Dickinson FACSCalibur System to assess the cell cycle phase distribution, with data analysis performed using FACS DIVA software.

#### 2.2.7 Western blot analysis

Western blot analysis was conducted on HeLa cells to investigate protein expression, following established methods. HeLa cells were treated with a range of lead compound concentrations (5–15 µM) for approximately 24 h, whereas control cells were incubated with only the complete media. Cells were lysed using a buffer containing a protease inhibitor cocktail (Roche). Post-lysis, proteins were resolved on an SDS-PAGE gel and then transferred to a nitrocellulose membrane using a Mini Trans-Blot Electrophoretic Transfer Cell (Bio-Rad). The membrane was incubated overnight with primary antibodies targeting p53, STAT3, and β-actin, followed by incubation with an HRP-conjugated secondary antibody. The blots were developed using DAB (Diaminobenzidine, Sigma, St. Louis, MO, United States) in the presence of peroxide.

#### 2.2.8 Statistical analysis

All data were presented as the mean ± standard deviation (n = 3). A dose–response relationship between the compounds was determined by running regression analyses on all of the data. Statistical significance was examined using Student’s t-test, and p < 0.05 was considered statistically significant.

## 3 Results

### 3.1 Identification of molecular target of natural compounds

Flavonoids, terpenoids, and phenolic compounds are known for their antiangiogenic effects, as supported by numerous phytochemical and pharmacological studies on various plants. *C. lanceolatus* is considered a potent antiangiogenic agent because it contains these compounds. However, detailed mechanisms or studies on the antiangiogenic effect of *C. lanceolatus* are yet to be determined.

A reverse pharmacophore mapping strategy was employed using *in silico* target screening with the PharmMapper server to identify bioactive compounds from *C. lanceolatus* that could serve as potential targets. As indicated in Supplementary Table S1, STAT3 was identified as one of the potential target candidates due to its high positive “Z” score. Although a high “Z”-score does not necessarily indicate irrelevance, a positive “Z”-score typically signifies a compound’s relevance to the target (Liu et al., 2010). Nineteen bioactive compounds in *C. lanceolatus* displayed a high positive “Z” score, suggesting they might interact with STAT3 and inhibit tumor cell proliferation. To corroborate the docking results, additional experimental studies are recommended to investigate the binding interactions of the bioactive compounds of *C. lanceolatus* with their therapeutic targets.

### 3.2 Binding modes predicted by docking

Molecular docking studies of the SH2 domain of STAT3 against 19 natural compounds from *C. lanceolatus* were performed. A shown in Table 2, 19 out of 38 compounds were selected for validation through docking studies. Autodock 4.2 was used to dock all compounds with the 3.25 Å resolution 3D X-ray crystal structure of the DNA-bound STAT3 homodimer (PDB ID: 1BG1) sourced from the Protein Data Bank. After the removal of the dimerisation partner of DNA and STAT3, the STAT3-SH2 domain’s binding area, containing active site amino acids (Met329- Phe710), was designated as the docking region. Hydrophobic interactions, hydrogen bonding, and π–π interactions, which are critical in protein–ligand complexes involving natural compounds, were identified as key interaction mechanisms. The orientation of inhibitors at the STAT3 active site was determined using Autodock 4.2, selecting the conformation with the best binding energy value for further analysis; these results are detailed in Table 2.

**TABLE 2:** Binding energy and specific interaction of STAT3 with compounds of *C. lanceolatus*.

Name of the compounds	Binding energy (kcal/mol)	Protein ligands interaction	
		Hydrophobic	H-bonds
Callislignan A	−6.1	Leu579, Asp570, Lys574	Arg335, Asp566
Callislignan B	−6.0	Pro471, Lys573, Asp566	Arg335
β-sitosterol	−4.9	Ala651, Phe683, Lys685	Arg335, Thr515
Ursolic acid	−4.0	Asn472, Trp474, Lys574	Arg335, Lys573, Thr515
Daucosterol	−5.4	Pro471, Trp474, Asp566	Arg335, Asp566, Lys573
Ellagic acid	−5.8	Leu579, Leu645, Tyr686	Arg335, Ile467, Ile576
α-lupenol	−4.4	Leu579, Asp566, Asp570	Arg335, Asp566 Asp570
Uvaol	−4.5	Asp570, Asp566	Arg335, Ser381, Lys383
Pelargonidin-3,5-diglucoside	−5.3	Thr575, Ser636, Thr641	Lys383, Pro471
Cyanidin-3,5-diglucoside	**−9.5**	Cys550, Glu552, Asn553	Arg335, Asp570, Lys573, Lys574, Glu616
Kaempferol	−5.7	Ile467, Asp566, Ile569	Arg335, Thr515
2 alpha –hydroxyl ursolic acid	−5.0	Thr341, Gln344, Met470	Lys573, Arg335
Quercetin	−5.6	Cys468, Met470,Lys573, Asp566	Arg335
kaempferol-3-o-β-d-galactopyranoside	**−8.9**	Ile467, Met470,Ile569,Val619,Thr641	Arg335, Lys573
quercetin-3-o-(2″-o-galloyl)-β-d-galactopyranoside	**−10.2**	Thr516, Trp562	His332, Arg33, Ile467, Asn567, Lys574, Glu616, Lys642
Betulic acid	−5.3	Lys658, Pro669	Arg335, Asp566
Alpha-amyrin	−5.4	Val338,Ile467,Cys468	Arg335, Thr515, Asp566
3,3′-di-O-methyl ellagic acid	−5.9	Ile467,Val338,Pro471	Arg335, Thr515
3,3′,4-tri-O-methyl ellagic acid	−6.0	His332, Ile467, Asp570	Arg335, Asp566
Known Inhibitor			
Plumbagin	**−6.2**	Asp570,Glu612,Lys615	Arg335, His332, Thr515
Sanguinarine	**−6.6**	Met331,Pro333,Glu415, Arg423	Arg335

The bold values indicate highest binding energy of ligands with protein.

**FIGURE 1:**
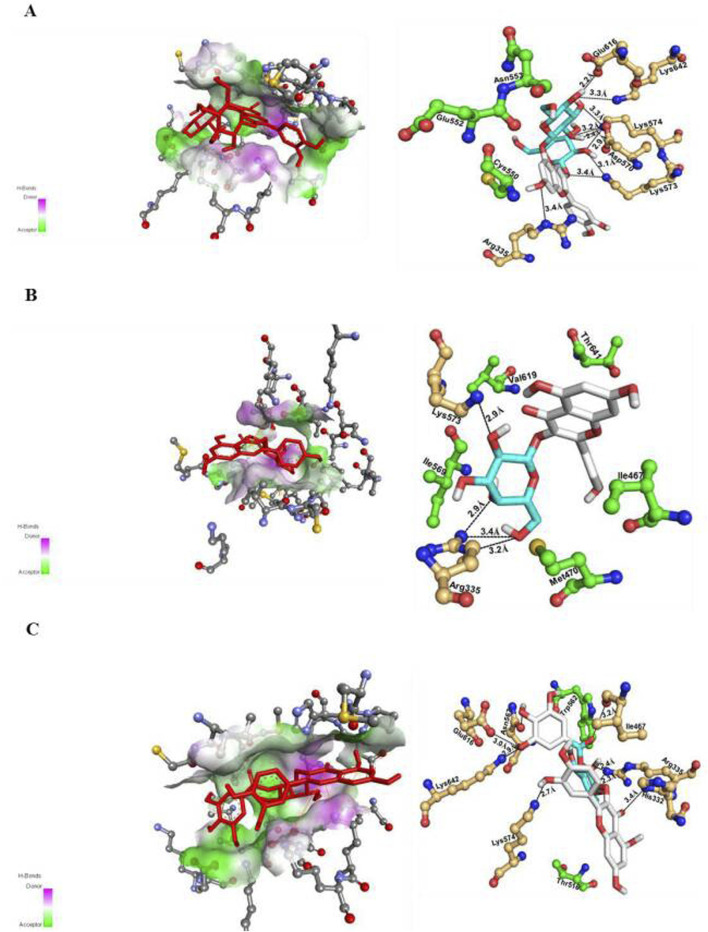
Docking pose of the promising compounds **(A)** cyanidin-3,5-diglucoside, **(B)** kaempferol-3-o-β-d-galactopyranoside and, **(C)** quercetin-3-o-(2″-o-galloyl)-β-d galactopyranoside along with key interactions and interacting amino acids at the active site of target STAT3 protein (PDB ID: 1BG1) are portrayed.

In this study, PyMOL software was used to analyse the binding modalities of STAT3 inhibitors to discover novel STAT3 inhibitors (Singh and Bast, 2015). The interactions depicted in Figure 1 were further examined using the STAT3 binding site.

The results demonstrated that cyanidin-3,5-diglucoside, kaempferol-3-o-β-d-galactopyranoside, and quercetin-3-o-(2″-o-galloyl)-β-d-galactopyranoside all bound effectively to the STAT3 active site, exhibiting minimum binding energies (∆G) of −9.5 kcal/mol, −8.9 kcal/mol, and −10.2 kcal/mol, respectively, compared with known STAT3 inhibitors, such as plumbagin and sanguinarine (Table 2).

The binding mechanism of quercetin-3-o-(2″-o-galloyl)-β-d-galactopyranoside, kaempferol-3-o-β-d-galactopyranoside, and cyanidin-3,5-diglucoside at the STAT3 active site differs significantly from that of plumbagin and sanguinarine. Cyanidin-3,5-diglucoside formed nine hydrogen bond interactions with Arg335, Asp570, Lys573, Lys574, and Glu616 (Figure 1A). Hydrophobic interactions were mediated by residues Cys550, Glu552, and Asn553, with π–π interactions involving Lys573, Lys574, and Arg335.

Kaempferol-3-o-β-d-galactopyranoside established nine hydrogen bonds with Arg335 and Lys573 (Figure 1B), with hydrophobic interactions facilitated by Ile467, Met470, Ile569, Val619, and Thr641 and π–π interactions stabilised by Lys574.

Quercetin-3-o-(2″-o-galloyl)-β-d-galactopyranoside formed eight hydrogen bonds with residues including His332, Arg335, Ile467, Asn567, Lys574, Glu616, and Lys642 (Figure 1C). Hydrophobic interactions involved Thr516 and Trp562, with π–π stabilisation by Lys573 and Lys574.

Cyanidin-3,5-diglucoside, kaempferol-3-o-β-d-galactopyranoside, and quercetin-3-o-(2″-o-galloyl)-β-d-galactopyranoside are considered potent STAT3 inhibitors due to their significant binding energy and interaction profiles.

### 3.3 MD simulations

**FIGURE 2:**
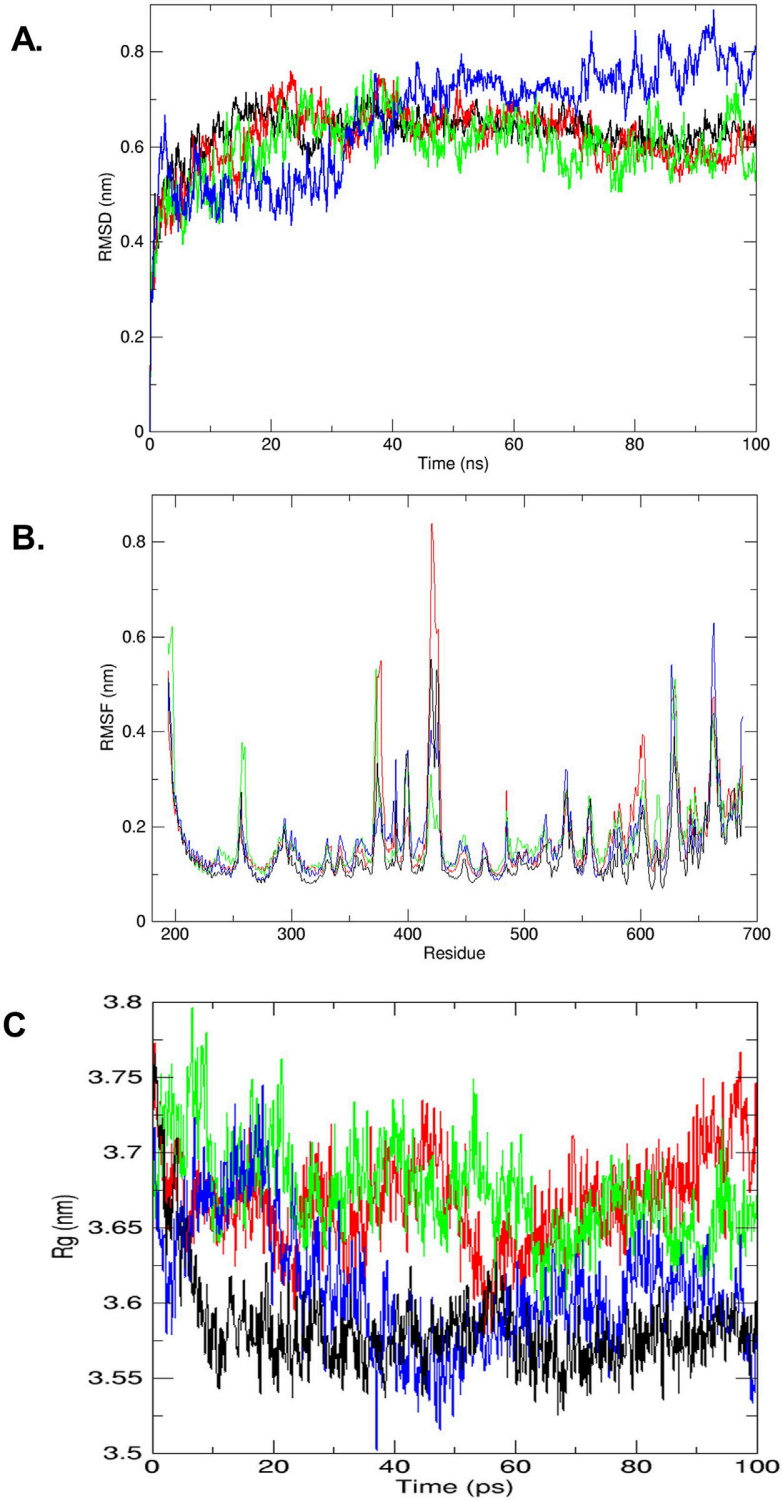
**(A)** RMSD plot of trajectories analyzed (by GROMACS), STAT3 receptor wild type (black) and complex of STAT3-compound cyanidin-3,5-diglucoside (red), STAT3-compound kaempferol-3-o-β-d-galactopyranoside (green) and STAT3-compound quercetin-3-o-(2″-o-galloyl)-β-d galactopyranoside (blue). **(B)** The trajectory analyze between RMSF (nm) vs. residues (by GROMACS), STAT3 receptor wild type (black) and complex of STAT3-compound cyanidin-3,5-diglucoside (red), STAT3-compound kaempferol-3-o-β-d-galactopyranoside (green) and STAT3-compound quercetin-3-o-(2″-o-galloyl)-β-d galactopyranoside (blue). **(C)** The trajectory analyze between Rg (nm) vs. time ns (by GROMACS), STAT3 receptor wild type (black) and complex of STAT3-compound cyanidin-3,5-diglucoside (red), STAT3-compound kaempferol-3-o-β-d-galactopyranoside (green) and STAT3-compound quercetin-3-o-(2″-o-galloyl)-β-d galactopyranoside (blue).

Protein stability and dynamics were key focal points in the MD simulation studies. The RMSD graph was employed to assess the behavior of the protein–ligand complexes over time, comparing the native protein (colored black) with complexes that include cyanidin-3,5-diglucoside, kaempferol-3-o-β-d-galactopyranoside, and quercetin-3-o-(2″-o-galloyl)-β-d-galactopyranoside. Figure 2A illustrates the RMSDs for the protein backbone of the wild type and the STAT3-cyanidin-3,5-diglucoside complex (colored red) over the simulation period. The RMSD values for cyanidin-3,5-diglucoside started at approximately 0.2 nm and increases to 0.7 between 20 ns and 40 ns, slowly stabilizing thereafter and reaching stabilizing at slightly higher than 0.6 nm at 100 ns around whereas the native protein’s RMSD began at approximately 0.2 nm and increased to around 0.65 nm. The kaempferol-3-o-β-d-galactopyranoside complex (colored green) exhibited greater fluctuations, starting at 5 ns and intensifying over time, with peak fluctuations observed around 0.7 nm between 20 and 40 ns and at around 95 ns. It reaches 0.5 nm at 100 ns while showing significant fluctuations during entire 100 ns. By contrast, the quercetin-3-o-(2″-o-galloyl)-β-d-galactopyranoside complex (colored blue) exhibited an increasing degree of fluctuation, showing significant variance, initially from 0 to 15 ns followed by periods of increasing fluctuations starting from 28 to 30 ns, the fluctuations continued till the 100 ns reaching 0.8 nm. It showed increased fluctuations after relatively more stable start., These findings indicate that overall cyanidin-3,5-diglucoside exhibits a lower RMSD and potentially greater stability than the native STAT3 protein, as highlighted by Khan et al. (2016), even though at 100 ns it has slightly higher RMSD value as compared to kaempferol-3-o-β-d-galactopyranoside. The latter showing more unpredictability as compared to the former.

The RMSF was used to assess the mobility of STAT3 amino acid residues (194–688) by tracking the trajectory of the Cα atom, which reflects the protein structure’s stability or flexibility. In the RMSF analysis, the STAT3-cyanidin-3,5-diglucoside complex (colored red) exhibited significant variations at residues 375, 425, 650 and 675 compared with the wild-type STAT3 (colored black). For the complex with kaempferol-3-o-β-d-galactopyranoside (colored green), major fluctuations were observed at residues 250-260, 375, 415-420, 600, 650-670. As shown in Figure 2B, the complex with quercetin-3-o-(2″-o-galloyl)-β-d-galactopyranoside (colored blue) demonstrated major fluctuations at residues 375-420 and 550-600, 625, 660-670.

The radius of gyration (Rg) plot was used to measure the compactness of the protein and its complexes with cyanidin-3,5-diglucoside, kaempferol-3-o-β-d-galactopyranoside, and quercetin-3-o-(2″-o-galloyl)-β-d-galactopyranoside. The Rg plot indicated that after 15 ns, the complex with quercetin-3-o-(2″-o-galloyl)-β-d-galactopyranoside decreased slightly and its lowest value at around 40 ns before gradually increasing till 80 ns before rapidly stabilizing at 100 ns reaching close to 3.525 nm suggesting enhanced stability compared with the other two complexes. By contrast, the complexes with cyanidin-3,5-diglucoside (colored red) and kaempferol-3-o-β-d-galactopyranoside (colored green) displayed initial decreases in Rg values while showing greater fluctuations thereafter which eventually aligned with the wild-type (colored black) level (Figure 2C).

### 3.4 ADME properties

Clinical trials for most drugs fail due to cellular toxicity and unfavourable pharmacokinetic properties. To assess the potential bioavailability of STAT3 inhibitors, the *in silico* pharmacokinetic profiles of the compounds of interest were analysed (Table 3). Drug absorption and bioavailability are inversely related to several physicochemical parameters, notably logS (water solubility), clogP (lipid solubility), MW (molecular weight), and polar surface area, which affect the drug’s release into the bloodstream from its delivery site (Van De Waterbeemd and Gifford, 2003; Mayank and Jaitak, 2016). Cytochrome P450 (CYP) enzymes play a critical role in drug metabolism, affecting the pharmacological, toxicological, and biological properties of drugs (de Graaf et al., 2005). In this study, the lead natural compounds from *C. lanceolatus* were evaluated for their pharmacokinetic and pharmacodynamic properties by using the ADMET tool in Discovery Studio 3.5. The compounds quercetin-3-o-(2″-o-galloyl)-β-d-galactopyranoside, kaempferol-3-o-β-d-galactopyranoside, and cyanidin-3,5-diglucoside exhibited the lowest binding energies. These compounds demonstrated favourable physicochemical characteristics, aligning with the criteria for drug-likeness, including lipophilicity (clogP), aqueous solubility (logS), polar surface area, and cytochrome P450 interactions (Table 3).

**TABLE 3:** Pharmacokinetics profile of known inhibitors and natural compounds.

ADMET										TOPKAT					
Compounds	BBB	AlogP	Sol.	HIA	HTL	HT_Prob	PPB	CYP2D6	PSA	Ames Mut.		Prob	Enrichment		WOE
Natural molecules															
Cyanidin-3,5-diglucoside	4	−0.912	3	0	0	0.53	1	0	277.93	NM		0.33	0.60		NC
Kaempferol-3-o-β-d-galactopyranoside	4	−0.057	3	3	1	0.88	2	1	189.79	NM		0.51	0.91		NC
Quercetin-3-o-(2″-o-galloyl)-β-d-galactopyranoside	4	1.018	3	3	1	0.93	1	0	278.46	NM		0.17	0.31		NC
Known Inhibitors															
Plumbagin	2	1.962	3	0	1	0.65	2	0	55.41	M		0.76	1.37		C
Sanguinarine	2	2.228	2	0	1	0.96	1	1	39.07	NM		0.70	1.25		NC

BBB: Blood Brain Barrier Level 0-4, having high penetration to no penetration, HIA: Human Intestinal Absorption level ideal value range from 0-1 as good to moderate, Sol. (Solubility Level): Ideal value of solubility level is 3, HTL: Hepatotoxicity level ideal value range from 0-1 as good to moderate, HepTox Prob.: Hepatotoxicity probability <0.5 is ideal, CYP2D6<0.5 is good and denoted with level 0, PPB: Plasma protein binding value 0 is good and compounds to accessible with BBB, AlogP value should not be greater than 5.0 and polar surface area ≤140 is ideal. Ames Mut: Ames mutagen prediction, Prob.: ames probability; Enrichment: Ames enrichment; WOE-_Prediction (weight of evidence); M: (Mutagen); NM: (Non-Mutagen); C: (Carcinogen); NC: (Non-Carcinogen).

### 3.5 QSTR analysis

In Table 3, the computed toxicity profiles for the compounds are presented. The initial step in applying a TOPKAT carcinogenicity predictor involves identifying structural similarities between the compounds being studied and reference molecules in the TOPKAT database (de Graaf et al., 2005). This study evaluated the mutagenic and carcinogenic potential of the compounds by using the weight of evidence (WOE) and Ames predictive models. Toxicological endpoints and models used in drug development cover a range of effects, including irritability, teratogenicity, sensitisation, neurotoxicity, and immunotoxicity. The selected compounds were found to be nonmutagenic, each having an Ames test likelihood score of seven or less. The WOE approach was used to assess the relative certainty of the compounds potentially causing cancer in humans. All compounds, with the exception of the sanguinarine inhibitors listed in Table 3, were deemed to be noncarcinogenic.

### 3.6 Biological activity spectrum

To evaluate the potential biological activity of some bioactive components, they were analysed using the PASS online service. The biological activity spectrum for each compound provides insights into potential pharmacological effects, action mechanisms, and specific toxicities. The probability values Pa (probability to be active) and Pi (probability to be inactive) are calculated independently, with their sum not exceeding 1, and their values ranging from 0 to 1. Pa values are indicative of activity, whereas Pi values suggest inactivity (Liao et al., 2013). Table 4 illustrates that the PASS prediction indicated higher Pa values than Pi values for the anti-neoplastic effects of the compounds.

**TABLE 4:** Biological activity spectrum of compounds (P_a_–Active; P_i_–Inactive).

.Name of the compounds	P_a_	P_i_	Activity
Cyanidin-3,5-diglucoside	0.829	0.049	Anti-neoplastic
Kaempferol-3-o-β-d-galactopyranoside	0.721	0.052	Anti-neoplastic
Quercetin-3-o-(2″-o-galloyl)-β-d-galactopyranoside	0.685	0.039	Anti-neoplastic

The antineoplastic properties of all three compounds were highlighted, with Pa values between 0.685 and 0.829. Among them, cyanidin-3,5-diglucoside exhibited the highest Pa value, suggesting a stronger antineoplastic potential than kaempferol-3-o-β-d-galactopyranoside and quercetin-3-o-(2″-o-galloyl)-β-d-galactopyranoside. These findings, supported by docking and simulation studies, suggest that these compounds can inhibit the STAT3 protein, which is instrumental in hindering angiogenesis, tumor growth, and metastatic progression.

### 3.7 Cytotoxicity studies

Metabolically active proliferating cells possess the enzyme succinate dehydrogenase, which reduces the yellow tetrazolium salt MTT to formazan, a process detailed in various studies (Alexandre et al., 2000; Badisa et al., 2006; Syarifah et al., 2011). The cytotoxicity and cell proliferation inhibitory effects of cyanidin-3,5-diglucoside, kaempferol-3-o-β-d-galactopyranoside, and quercetin-3-o-(2″-o-galloyl)-β-d-galactopyranoside, derived from *C. lanceolatus*, were evaluated using the MTT assay. This assay was conducted on the HEK-293 cell line to determine cytotoxicity. Supplementary Figure S1a illustrates the cell viability before and after treatment with these compounds, using coumarin as a control. The HEK293 cells showed no toxicity at concentrations up to 200 μM for cyanidin-3,5-diglucoside, kaempferol-3-o-β-d-galactopyranoside, and quercetin-3-o-(2″-o-galloyl)-β-d-galactopyranoside. The IC50 values for these compounds against HEK-293 cells were 151.55 µM, 146.62 µM, and 175.96 µM, respectively, when compared with the standard (Table 5).

**TABLE 5:** IC_50_ (µM) and selectivity index (SI) values of *C. lanceolatus* derived natural compounds on HEK293, HeLa and MCF7 cell lines.

Compounds	HEK293	HeLa		MCF7	
	IC_50_(µM)±S.D.	IC_50_(µM)±S.D.	SI	IC_50_(µM)±S.D.	SI
Cyanidin-3,5-diglucoside	151.55 ± 1.36	13.84 ± 1.51	11.23	53.37 ± 1.45	2.83
Kaempferol-3-o-β-d-galactopyranoside	146.62 ± 1.58	15.86 ± 1.67	9.24	76.83 ± 1.61	1.90
Quercetin-3-o-(2″-o-galloyl)-β-d-galactopyranoside	175.96 ± 1.55	09.93 ± 1.52	17.72	61.46 ± 1.48	2.86
Coumarin	138.29 ± 1.36	19.58 ± 1.01	7.06	24.51 ± 1.89	5.64

When assessing their anticancer activity against HeLa and MCF7 cell lines, cell viability significantly decreased in a dose-dependent manner. Supplementary Figure S1B displays the results of the cell proliferation assay for the HeLa cell line using cyanidin-3,5-diglucoside, kaempferol-3-o-β-d-galactopyranoside, quercetin-3-o-(2″-o-galloyl)-β-d-galactopyranoside, and coumarin as the reference compound. IC50 values were calculated for each compound based on the percentage of cell viability data (Table 5). The compounds quercetin-3-o-(2″-o-galloyl)-β-d-galactopyranoside, kaempferol-3-o-β-d-galactopyranoside, and cyanidin-3,5-diglucoside demonstrated the lowest IC50 values, suggesting higher efficacy against HeLa cells.

Supplementary Figure S1C displays the results of a cell proliferation assay evaluating the impact of several substances on MCF7 cells, including cyanidin-3,5-diglucoside, kaempferol-3-o-β-d-galactopyranoside, and quercetin-3-o-(2″-o-galloyl)-β-d-galactopyranoside, with coumarin serving as the reference compound. The IC50 values listed in Table 5 six suggest that these substances exhibit decreased efficacy against MCF7 cancer cells.

Many compounds derived from *C. lanceolatus* had their SI values calculated for their effectiveness against malignant cells. Cancer cells were selectively inhibited by extracts with a high SI value (>3). Normal cells were considered to be toxic with an SI value less than 3 (Badisa et al., 2006). Table 5 indicates that the compounds cyanidin-3,5-diglucoside, kaempferol-3-o-β-d-galactopyranoside, and quercetin-3-o-(2″-o-galloyl)-β-d-galactopyranoside exhibited good selectivity against HeLa cell lines. For MCF7 cell lines, quercetin-3-o-(2″-o-galloyl)-β-d-galactopyranoside, kaempferol-3-o-β-d-galactopyranoside, and cyanidin-3,5-diglucoside had SI values less than 3.

The results were supported by those of DAPI, cell migration, cell cycle analysis, mRNA and protein expression data, indicating that cyanidin-3,5-diglucoside, kaempferol-3-o-β-d-galactopyranoside, and quercetin-3-o-(2″-o-galloyl)-β-d-galactopyranoside are selective and more active against HeLa cells than MCF7 cells.

### 3.8 Fluorescence microscopic analysis of cell death

**FIGURE 3:**
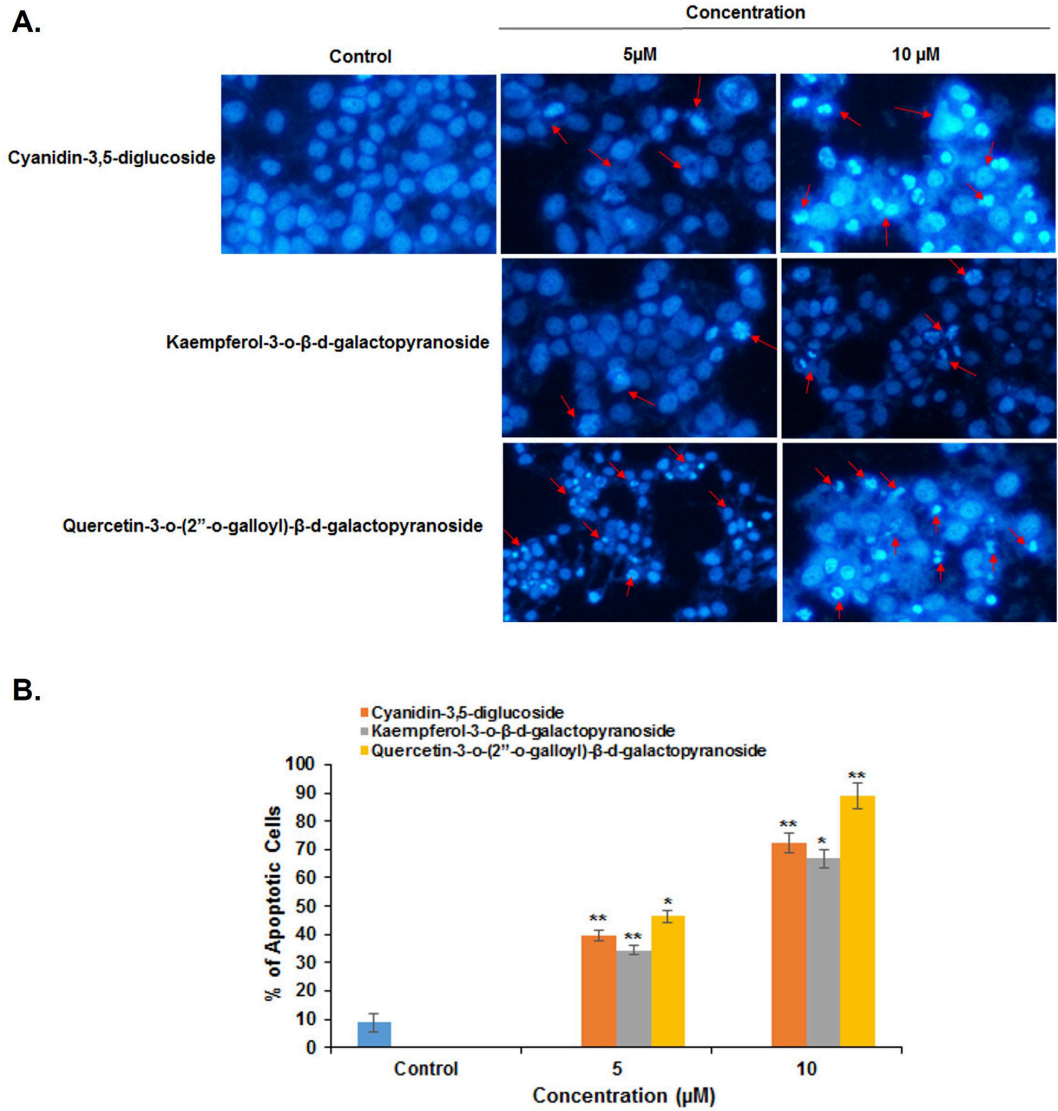
**(A)** HeLa cancer cells were stained with DAPI without treatment compounds (Control) and treated with different compounds, **(B)** Histogram representation of the percentage of apoptotic cells against their different concentrations of compounds used to treat HeLa cells. *p < 0.05; **p < 0.01 compared with the control.

Fluorescence microscopy (Nikon Eclipse, Inc., Japan) was used to differentiate between necrotic and apoptotic cells by examining their morphology and cell membrane integrity. In a fluorescence microscopy analysis, untreated HeLa cells displayed uniform oval shapes and uniformly blue fluorescence. After 48 h of treatment with cyanidin-3,5-diglucoside, kaempferol-3-o-β-d-galactopyranoside, and quercetin-3-o-(2″-o-galloyl)-β-d-galactopyranoside at concentrations of 5 µM and 10 μM, varied cellular morphologies were observed (Figure 3A).

The average numbers and standard deviations of apoptotic cells were calculated. At 5 µM and 10 μM, cyanidin-3,5-diglucoside, kaempferol-3-o-β-d-galactopyranoside, and quercetin-3-o-(2″-o-galloyl)-β-d-galactopyranoside significantly increased the number of apoptotic cells (Figure 3B).

### 3.9 *In vitro* wound-healing assay (cell migration assay)

**FIGURE 4:**
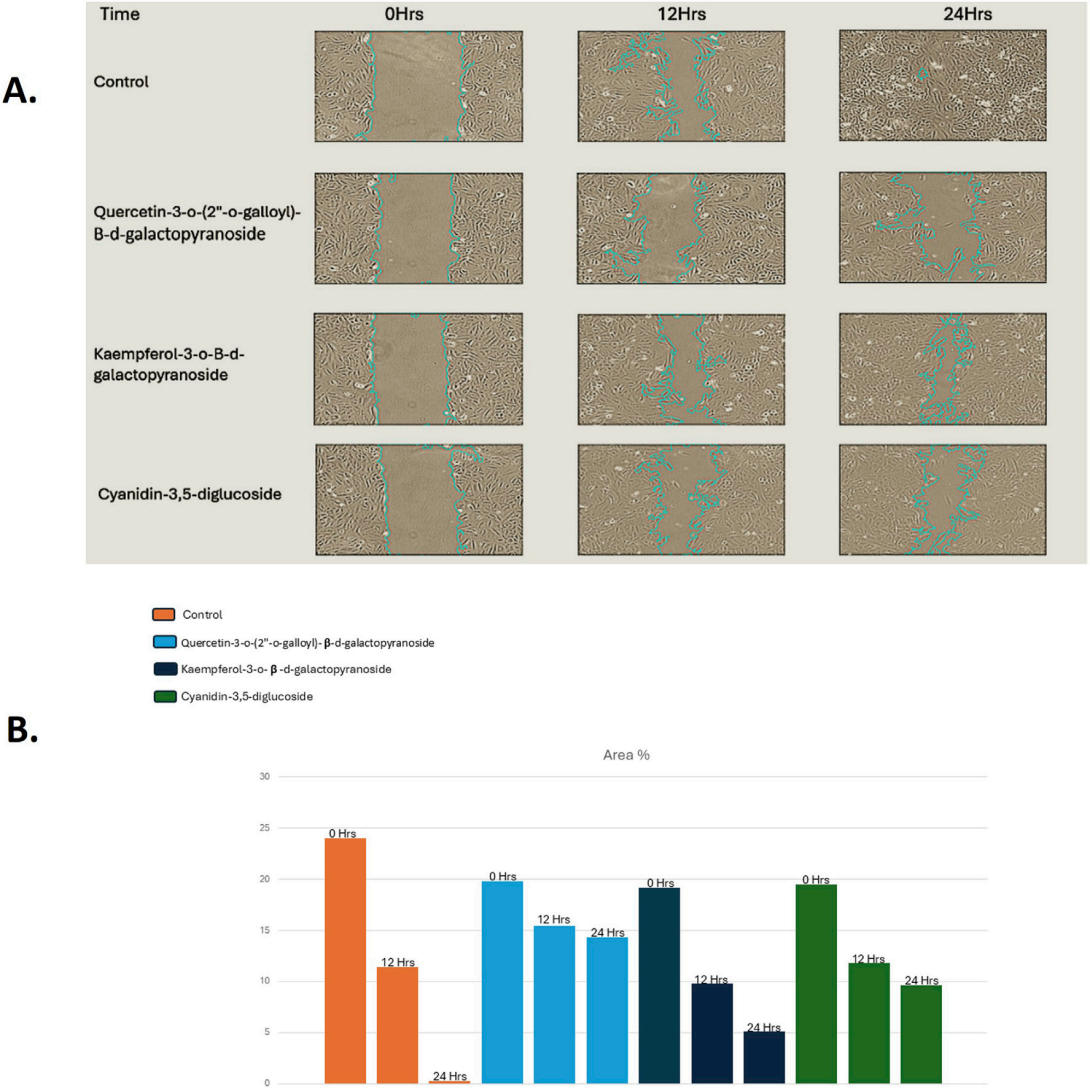
**(A)** Width of wound increases effectively after the treatment of compounds for 48 h in HeLa cell lines as compared without treatment compounds (Control), **(B)** Graph representation of the percentage of wound against their different concentrations for compounds treated Hela cancer cells as compared to Control.

The wound-healing assay showed that at concentrations of 5 µM and 10 μM, the compounds quercetin-3-o-(2″-o-galloyl)-β-d-galactopyranoside, kaempferol-3-o-β-d-galactopyranoside, and cyanidin-3,5-diglucoside significantly reduced HeLa cell migration. Figure 4A presents representative photomicrographs of HeLa cells after treatment with different concentrations of these compounds.

Figure 4B illustrates the wound closure rate, quantified from images obtained 48 h post-treatment; scale bars were added to all images using ImageJ for clarity. The figures reveal that the wound width increased after 48 h of treatment with cyanidin-3,5-diglucoside, kaempferol-3-o-β-d-galactopyranoside, and quercetin-3-o-(2″-o-galloyl)-β-d-galactopyranoside. Figures 4A, B indicate that, relative to the untreated control, these compounds exhibited a more pronounced reduction in cell migration.

### 3.10 Effect of compounds on cell cycle regulation

**FIGURE 5:**
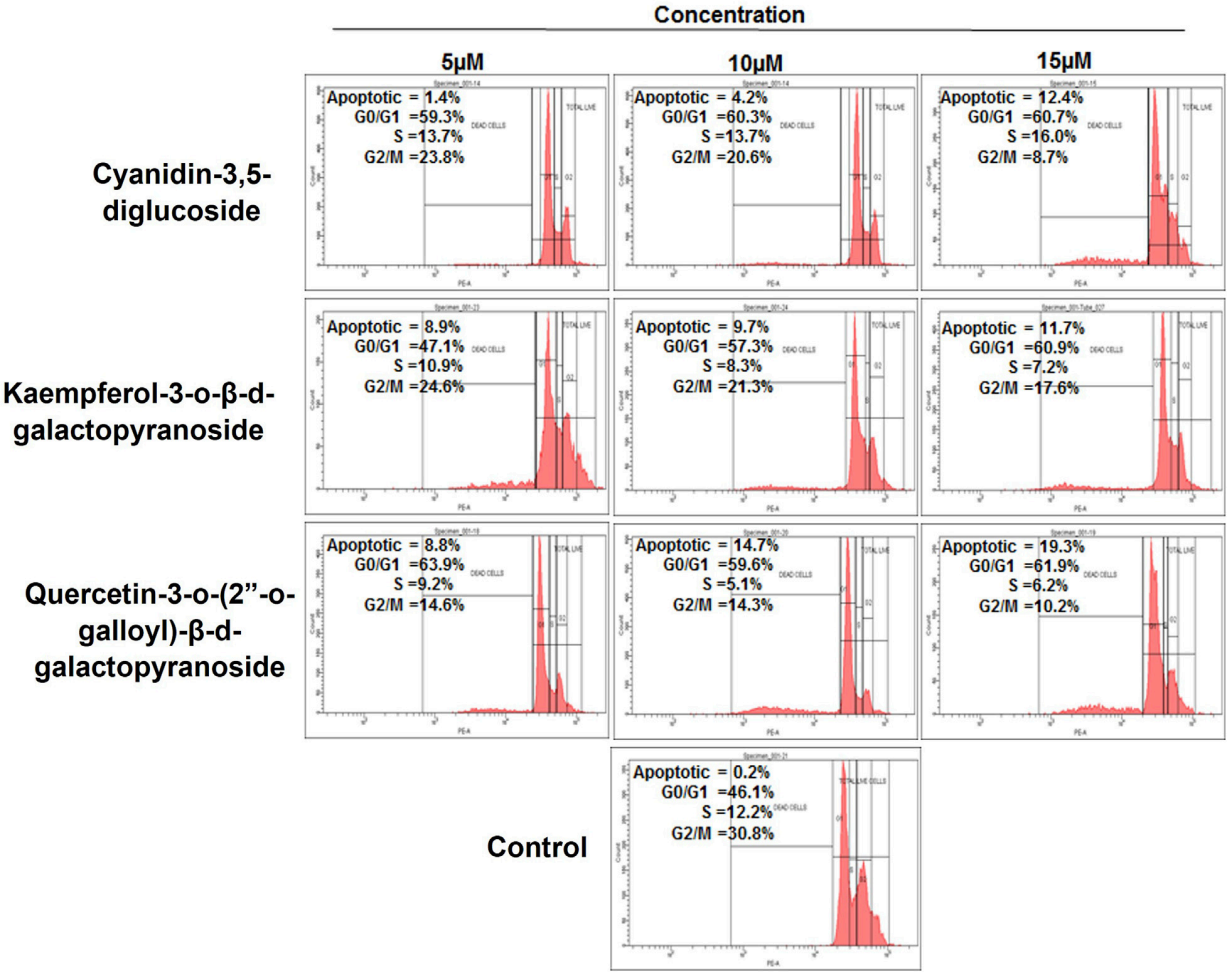
Cell cycle phase distribution against HeLa cells with treatment of compounds as compared to control.

The compounds cyanidin-3,5-diglucoside, kaempferol-3-o-β-d-galactopyranoside, and quercetin-3-o-(2″-o-galloyl)-β-d-galactopyranoside were analysed using flow cytometry combined with propidium iodide labelling to assess their ability to modulate the cell cycle in HeLa cells. Figure 5 illustrates the proportion of cells that underwent apoptosis and their cell cycle phase after 48 h of treatment with these compounds. The exposure to varying concentrations of cyanidin-3,5-diglucoside resulted in apoptosis rates of 1.4%, 4.2%, and 12.4% in the HeLa cell line, in contrast to the untreated control group’s rate of 0.2%.

Figure 5 shows that HeLa cells treated with 5, 10, and 15 µM of kaempferol-3-o-β-d-galactopyranoside experienced apoptosis in 8.9%, 9.7%, and 11.7% of cells, respectively, compared with the control group’s rate of 0.2%. Similarly, treatment with quercetin-3-o-(2″-o-galloyl)-β-d-galactopyranoside at doses of 5 μM, 10 μM, and 15 µM led to apoptosis rates of 8.8%, 14.7%, and 19.3%, respectively, against a control apoptosis rate of 0.2%.

HeLa cells exhibited an increase in G0/G1 phase after 48 h of treatment with cyanidin-3,5-diglucoside, kaempferol-3-o-d-galactopyranoside, and quercetin-3-o-(2″-o-galloyl)-β-d-galactopyranoside at dosages of 5, 10, and 15 µM. The control group did not show any increase. Compared with other chemicals presented in Figure 5, cyanidin-3,5-diglucoside accompanied this increase with a higher percentage of cells in S phase. Cyanidin-3,5-diglucoside, kaempferol-3-o-β-d-galactopyranoside, and quercetin-3-o-(2″-o-galloyl)-β-d-galactopyranoside reduced the percentage of G2/M treated cells compared with controls.

### 3.11 Compound effects on STAT3 and P53 mRNA expression in HeLa cancer cells

**FIGURE 6:**
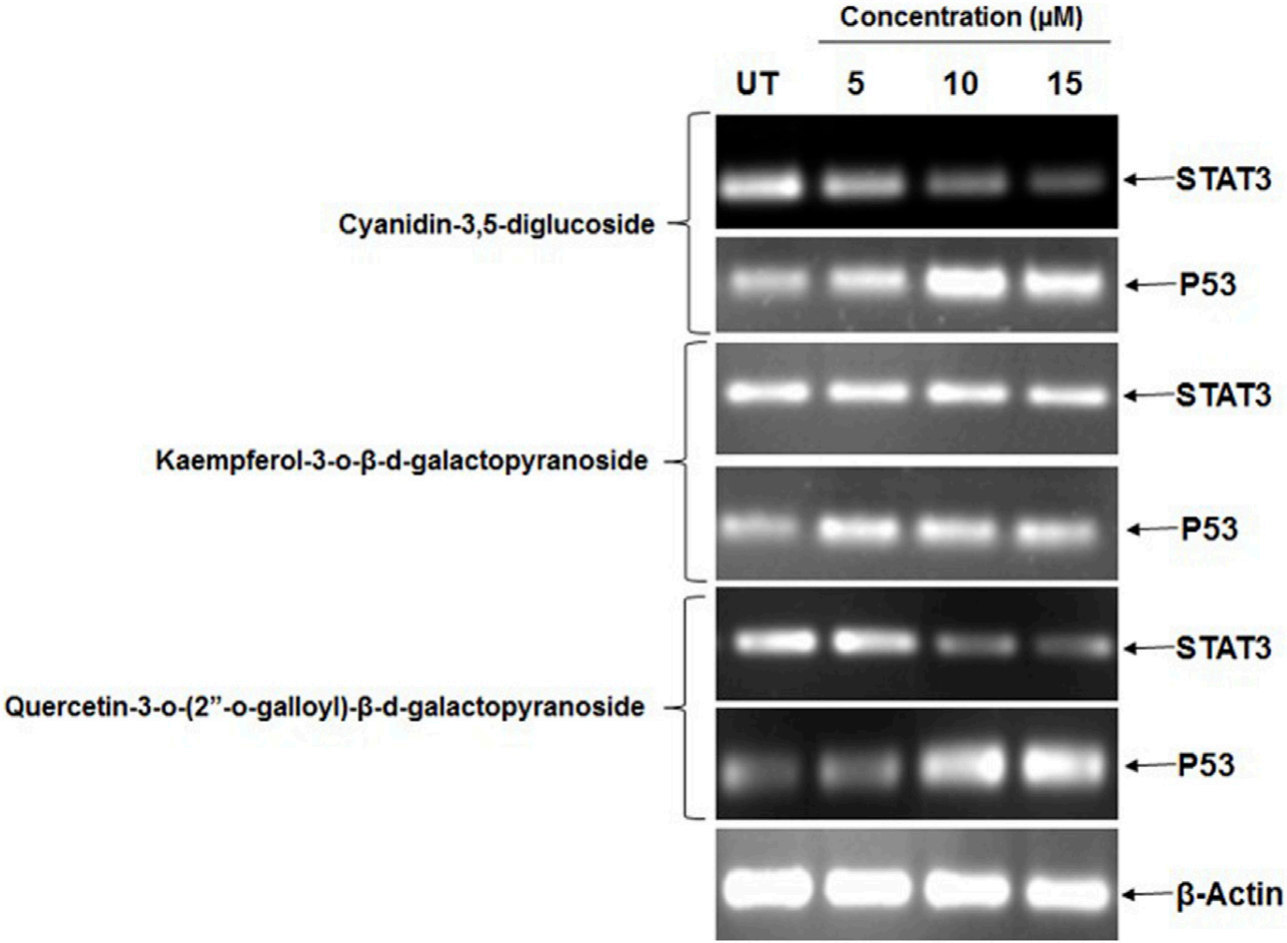
HeLa cells showed a marked decrease in mRNA expression of STAT3 and increase in mRNA expression of p53 after the treatment of different concentration of natural compounds. Control (UT).

In this study, the effects of cyanidin-3,5-diglucoside, kaempferol-3-o-β-d-galactopyranoside, and quercetin-3-o-(2″-o-galloyl)-β-d-galactopyranoside on STAT3 expression in HeLa cells were evaluated. Additionally, the effect of these substances on p53 mRNA expression in HeLa cells was investigated. After a 48-h incubation with these compounds, mRNA expression levels were assessed through RT-PCR. The RT-PCR results indicated that these substances possess STAT3 inhibitory potential, leading to increased p53 expression. Figure 6 demonstrates that at 5 μM, 10 μM, and 15 µM concentrations, there was a decrease in STAT3 mRNA expression and an increase in p53 mRNA expression in HeLa cells.

### 3.12 Effects of compounds on the levels of STAT3 and p53 protein in HeLa cells

**FIGURE 7:**
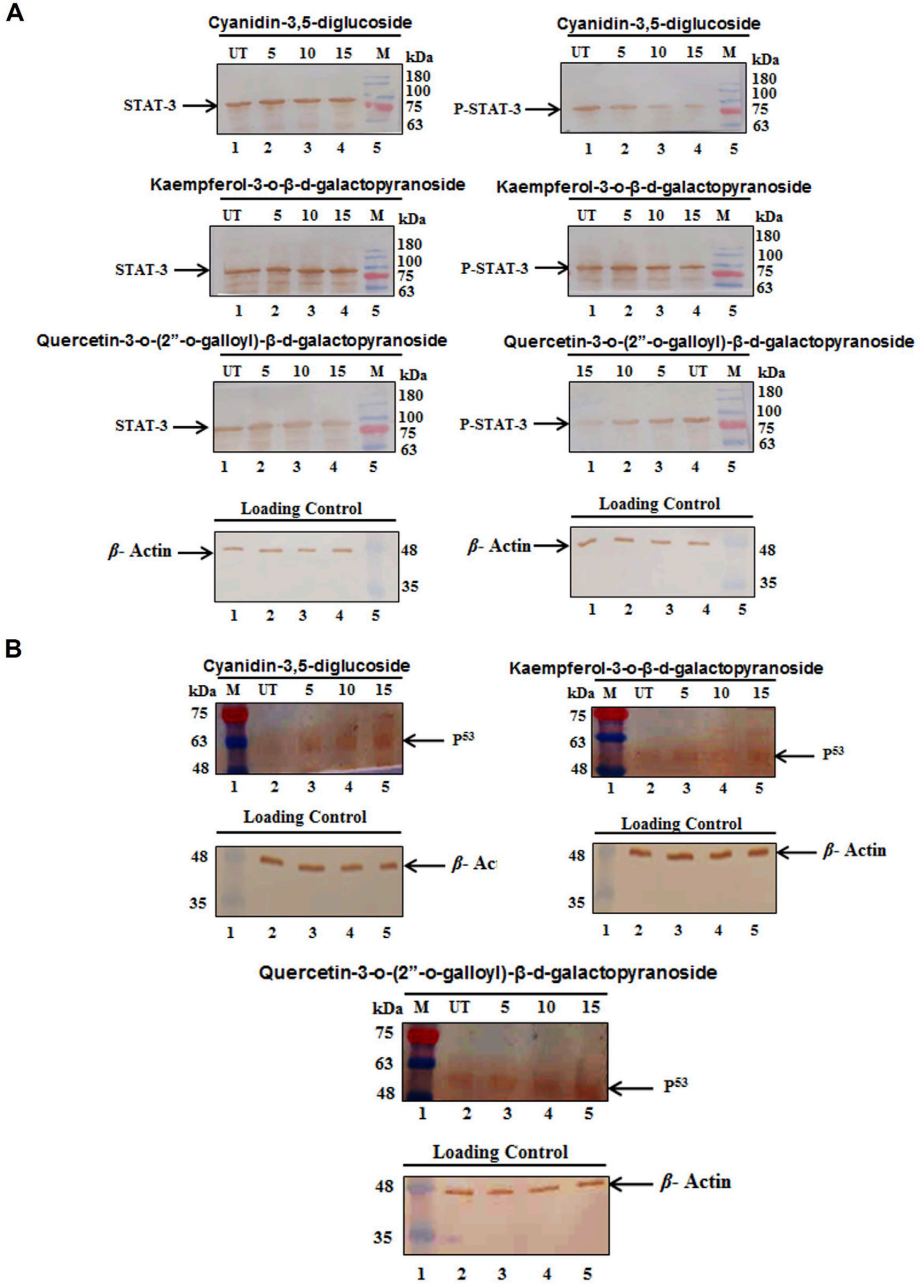
HeLa cells showed **(A)** a marked decrease in protein expression of pSTAT3 but the level of STAT3 was not changed and **(B)** a marked increase in protein expression of p53 after the treatment of different concentration of compounds. Control (UT), Marker (M).

The study explored how these compounds induce G0/G1 arrest in HeLa cells by modulating molecular pathways involving the suppression of STAT3 and an increase in p53 protein levels. The results of Western blot assays for STAT3 and p53 proteins are shown in Figure 7. The findings indicate that treatment with cyanidin-3,5-diglucoside, kaempferol-3-o-β-d-galactopyranoside, and quercetin-3-o-(2″-o-galloyl)-β-d-galactopyranoside increased p53 levels and reduced p-STAT3 levels in HeLa cells, whereas total STAT3 levels remained constant (Figures 7A, B).

## 4 Discussion

From the previous studies, *C. lanceolatus* is known to be contain potent antiangiogenic agent as Flavonoids, terpenoids, and phenolic compounds are extracted from it for cancer therapy (Ahmed et al., 2018). This study identified a naturally occurring compound from *C. lanceolatus* which is known to have many biological properties (Kumar et al., 2011) that effectively binds to STAT3, inhibits its translocation and DNA binding, and ultimately prevents STAT3-dependent cancer transformation.

To further establish its role the targets for these agents were analyzed using PharmMapper server, we found STAT3 as a potential target with high ‘Z’ score, 19 bioactive compounds seem to have positive ‘Z’ score and seems to have interacted with STAT3.

To further corroborate this interaction these nineteen bioactive compounds and SH2 domain of STAT3 protein was undergone through molecular docking using Autodock 4.2. The result suggested that cyanidin-3,5-diglucoside, kaempferol-3-o-β-d-galactopyranoside, and quercetin-3-o-(2″-o-galloyl)-β-d-galactopyranoside, derived from *C. lanceolatus*, as potential STAT3 inhibitors (Table 2). The molecular docking results revealed hydrophobic interactions, hydrogen bonds, and π–π interactions as primary forces in the binding of these compounds to the SH2 domain of STAT3. These compounds exhibited promising drug-like properties, as indicated by their ADME, QSTR profiles, and biological activity spectra analyses. All these compounds were seemed to have lowest binding energy as an inhibitor of STAT3 as compared to other known inhibitors such as plumbagin and sanguinarine (Bhat et al., 2024).

The MD simulation studies were performed to assess the protein and ligand complex stability and dynamics via RMSD graph (Figure 2A). The results from all three ligands indicate that cyanidin-3,5-diglucoside exhibits a lower RMSD and potentially greater stability than the native STAT3 protein, as highlighted by Khan et al. (2016).

Further, the RMSF was used to assess the mobility of STAT3 amino acid residues (194–688) by tracking the trajectory of the Cα atom, which reflects the protein structure’s stability or flexibility here we found that the STAT3-cyanidin-3,5-diglucoside complex exhibited minor variations at residues 425 and 535–536 compared with the wild-type STAT3 (Figure 2B). Also, the radius of gyration (Rg) plot was used to measure the compactness of the protein and its complexes with these compounds. The results have shown that the complex with quercetin-3-o-(2″-o-galloyl)-β-d-galactopyranoside decreased slightly and then stabilised, suggesting enhanced stability compared with the other two complexes (Figure 2C).

Clinical trials for most drugs fail due to cellular toxicity and unfavourable pharmacokinetic properties. To assess the potential bioavailability of STAT3 inhibitors, the *in silico* pharmacokinetic profiles of the compounds of interest were analysed (Table 3). Drug absorption and bioavailability are inversely related to several physicochemical parameters, notably logS (water solubility), clogP (lipid solubility), MW (molecular weight), and polar surface area, which affect the drug’s release into the bloodstream from its delivery site (Van De Waterbeemd and Gifford, 2003; Mayank and Jaitak, 2016). Cytochrome P450 (CYP) enzymes play a critical role in drug metabolism, affecting the pharmacological, toxicological, and biological properties of drugs (de Graaf et al., 2005).

In our study by using ADMET tool in Discovery Studio 3.5, our compounds have shown favourable physicochemical characteristics, aligning with the criteria for drug-likeness, including lipophilicity (clogP), aqueous solubility (logS), polar surface area, and cytochrome P450 interactions.

In Table 3, the computed toxicity profiles for the compounds are presented. The initial step in applying a TOPKAT carcinogenicity predictor involves identifying structural similarities between the compounds being studied and reference molecules in the TOPKAT database (de Graaf et al., 2005). This study evaluated the mutagenic and carcinogenic potential of the compounds by using the weight of evidence (WOE) and Ames predictive models. Toxicological endpoints and models used in drug development cover a range of effects, including irritability, teratogenicity, sensitisation, neurotoxicity, and immunotoxicity. The selected compounds of our study were found to be non-mutagenic.

The biological activity spectrum for each compound provides insights into potential pharmacological effects, action mechanisms, and specific toxicities. The probability values Pa (probability to be active) and Pi (probability to be inactive) are calculated independently, with their sum not exceeding 1, and their values ranging from 0 to 1. Pa values are indicative of activity, whereas Pi values suggest inactivity (Liao et al., 2013). Table 4 illustrates that the PASS prediction indicated higher Pa values than Pi values for the anti-neoplastic effects of the compounds. Cyanidin-3,5-diglucoside exhibited the highest Pa value, suggesting a stronger antineoplastic potential than kaempferol-3-o-β-d-galactopyranoside and quercetin-3-o-(2″-o-galloyl)-β-d-galactopyranoside.

All these results indicate that these compounds inhibit the STAT3 protein hindering the tumor growth and cancer progression.

Metabolically active proliferating cells possess the enzyme succinate dehydrogenase, which reduces the yellow tetrazolium salt MTT to formazan, a process detailed in various studies (Alexandre et al., 2000; Badisa et al., 2006; Syarifah et al., 2011). The cytotoxicity and cell proliferation inhibitory effects of cyanidin-3,5-diglucoside, kaempferol-3-o-β-d-galactopyranoside, and quercetin-3-o-(2″-o-galloyl)-β-d-galactopyranoside, derived from *C. lanceolatus*, were evaluated using the MTT assay on normal cell line HEK293 we found that no toxicity at concentrations up to 200 μM for these compounds were seen (Table 5).

The anticancer activity of the compounds was assessed on MCF-7 and HeLa cell line and we found that these compounds have higher efficacy towards HeLa cells with lower IC50 values.

Many compounds derived from *C. lanceolatus* had their SI values calculated for their effectiveness against malignant cells. Cancer cells were selectively inhibited by extracts with a high SI value (>3). Normal cells were considered to be toxic with an SI value less than 3 (Badisa et al., 2006). Cyanidin-3,5-diglucoside, kaempferol-3-o-β-d-galactopyranoside, and quercetin-3-o-(2″-o-galloyl)-β-d-galactopyranoside exhibited good selectivity against HeLa cell lines whereas, for MCF-7 the value is greater than 3.

The results were supported by those of DAPI, cell migration, cell cycle analysis, mRNA and protein expression data, indicating that cyanidin-3,5-diglucoside, kaempferol-3-o-β-d-galactopyranoside, and quercetin-3-o-(2″-o-galloyl)-β-d-galactopyranoside are selective and more active against HeLa cells than MCF7 cells.

To study the effect of these compounds on cell migration, one of the major events in cancer progression we treated HeLa cells with different concentration of compounds and found reduction in cell migration. This was achieved through wound healing assay.

To further establish the effect of compounds on cancer cells we analysed its role in cell cycle regulation through flow cytometry and found that cyanidin-3,5-diglucoside affected cell cycle arrest and cell accumulation in the G0/G1 and S phases, whereas compounds kaempferol-3-o-β-d-galactopyranoside and quercetin-3-o-(2″-o-galloyl)-β-d-galactopyranoside affected cell cycle arrest and cell accumulation in the G0/G1 phases (Figure 5). The results suggested that cyanidin-3,5-diglucoside, kaempferol-3-o-β-d-galactopyranoside, and quercetin-3-o-(2″-o-galloyl)-β-d-galactopyranoside caused apoptosis in HeLa cells by arresting the G0/G1 cell cycle.

The lack of p53 suppression in cancer cells leads to unregulated cell division due to the noninhibition of the cell cycle’s CDKs (Molinari, 2000; Niu et al., 2005; Giono and Manfredi, 2006).

Our RT-PCR results indicate that these substances possess STAT3 inhibitory potential, leading to increased p53 expression. Figure 6 demonstrates that at 5 μM, 10 μM, and 15 µM concentrations, there was a decrease in STAT3 mRNA expression and an increase in p53 mRNA expression in HeLa cells. The protein expression was done using Western blot analysis and it coincided with mRNA expression, the downregulation of pSTAT3 and upregulation of p53 suggest that p53 inhibits CDK, promoting G0/G1 arrest in the cell cycle, with cyanidin-3,5-diglucoside uniquely inducing S phase arrest (Niu et al., 2005).

All these activities indication potential of these compounds as anti-proliferative agent in cancer cells, to concrete these findings, further investigation into natural compounds derived from *C. lanceolatus* as targeting agents against STAT3 is suggested by an *in silico* research. Experimental events, such as cytotoxicity, antiproliferative activity, DAPI, cell migration, cell cycle analysis, and mRNA and protein expression results on STAT3 and p53 bolster the possibility of investigating these compounds as STAT3-targeting agents.

## 5 Conclusion

This study identified natural compounds from *C. lanceolatus* that inhibit STAT3, demonstrating suppressive effects on cervical cancer cell lines and highlighting their anticancer properties. Research pinpointed STAT3 inhibitors within *C. lanceolatus*, specifically cyanidin-3,5-diglucoside, kaempferol-3-o-β-d-galactopyranoside, and quercetin-3-o-(2″-o-galloyl)-β-d-galactopyranoside, using molecular docking and simulation studies.

*C. lanceolatus* compounds were found to inhibit cell proliferation and migration, with notable reductions in cell growth observed after 48 h of treatment. Also, natural compounds from *C. lanceolatus* decrease STAT3 mRNA levels while increasing p53 mRNA expression. These findings suggest that these compounds from *C. lanceolatus* can inhibit STAT3 protein activity, reducing cervical cancer cell proliferation and offering potential applications in cancer chemoprevention and therapy in near future.

Our study can contribute to the field of Chemistry by elucidating the chemical nature of natural compounds from *C. lanceolatus* and their potential as anticancer agents targeting STAT3 pathway. The findings offer valuable information for the design and optimization of future drug candidates with improved efficacy and reduced toxicity profiles and further helps in alternative to chemo-drugs which are known to show resistance in cancer patients.

## Data Availability

The original contributions presented in the study are included in the article/Supplementary Material, further inquiries can be directed to the corresponding author.
