# Supplementary material for: Elucidation of Callistemon lanceolatus-derived natural compounds in STAT 3 pathway against human cancer cells: in silico and in vitro studies
Source: Front Pharmacol. 2025 Feb 21;16:1507002. doi: 10.3389/fphar.2025.1507002 (PMC11885254; doi:10.3389/fphar.2025.1507002)
Supplement: Supplementary file 1 [file Table1.docx]

**Supplementary Table 1:** The possibility of STAT3 target identification by reverse pharmacophore mapping approach

| **Name of the compounds** | **Possibility for STAT 3** | **Fit score** | **Normalized fit score** | **‘Z’ score** |
| --- | --- | --- | --- | --- |
| Callislignan A | Yes | 4.29 | 0.613 | 1.035 |
| Callislignan B | Yes | 4.59 | 0.353 | 0.979 |
| β-sitosterol | Yes | 4.33 | 0.619 | 0.855 |
| ursolic acid | Yes | 4.15 | 0.436 | 1.377 |
| daucosterol | Yes | 4.07 | 0.396 | 1.225 |
| gallic acid | No | 3.73 | 0.534 | -0.719 |
| ellagic acid | Yes | 4.45 | 0.445 | 1.225 |
| α-lupenol | Yes | 4.29 | 0.613 | 1.055 |
| uvaol | Yes | 4.25 | 0.607 | 1.065 |
| α-Pinene | No | 3.72 | 0.531 | -1.069 |
| α-Phellandrene | No | 3.87 | 0.430 | -1.054 |
| D-Limonene | No | 3.86 | 0.322 | -1.062 |
| 1,8-cineole | No | 3.86 | 0.351 | -1.065 |
| α-Terpineol | No | 3.88 | 0.323 | -1.054 |
| α-Thujene | No | 3.73 | 0.467 | -1.672 |
| Spathulenol | No | 4.24 | 0.385 | -0.028 |
| Pelargonidin-3,5-diglucoside | Yes | 4.17 | 0.632 | 2.055 |
| Cyanidin-3,5-diglucoside | Yes | 4.98 | 0.891 | 2.413 |
| kaempferol | Yes | 3.98 | 0.386 | 1.195 |
| 2 alpha –hydroxyl ursolic acid | Yes | 4.15 | 0.448 | 1.578 |
| Gallic acid 4-o-methyl ester | No | 4.07 | 0.508 | -0.054 |
| Quercetin | Yes | 4.14 | 0.690 | 0.015 |
| kaempferol-3-o-β-d-galactopyranoside | Yes | 5.23 | 0.980 | 2.725 |
| quercetin-3-o-(2”-o-galloyl)-β-d-galactopyranoside | Yes | 5.19 | 0.952 | 2.696 |
| Betulic acid | Yes | 4.05 | 0.579 | 0.038 |
| Alpha-amyrin | Yes | 4.17 | 0.575 | 2.031 |
| Pyrogallol | No | 4.35 | 0.622 | -0.014 |
| catechol | No | 4.09 | 0.454 | -0.014 |
| 3,3΄-di-O-methyl ellagic acid | Yes | 3.91 | 0.782 | 0.053 |
| 3,3΄,4-tri-O-methyl ellagic acid | Yes | 3.99 | 0.799 | 0.048 |
| Eugenol | No | 3.72 | 0.465 | -1.435 |
| Geraniol | No | 3.72 | 0.531 | -1.485 |
| Hydrocinnamyl acetate | No | 3.81 | 0.423 | -1.413 |
| Leptospermone | No | 3.80 | 0.543 | -1.357 |
| Methyl eugenol | No | 3.73 | 0.467 | -1.672 |
| Flavesone | No | 3.77 | 0.419 | -1.410 |
| Eugenol acetate | No | 3.73 | 0.533 | -1.421 |
| Dihydroxydurene | No | 3.73 | 0.339 | -1.634 |
