# Supplementary material for: Elucidation of Callistemon lanceolatus-derived natural compounds in STAT 3 pathway against human cancer cells: in silico and in vitro studies
Source: Front Pharmacol. 2025 Feb 21;16:1507002. doi: 10.3389/fphar.2025.1507002 (PMC11885254; doi:10.3389/fphar.2025.1507002)
Supplement: Supplementary file 2 [file Image1.pdf]

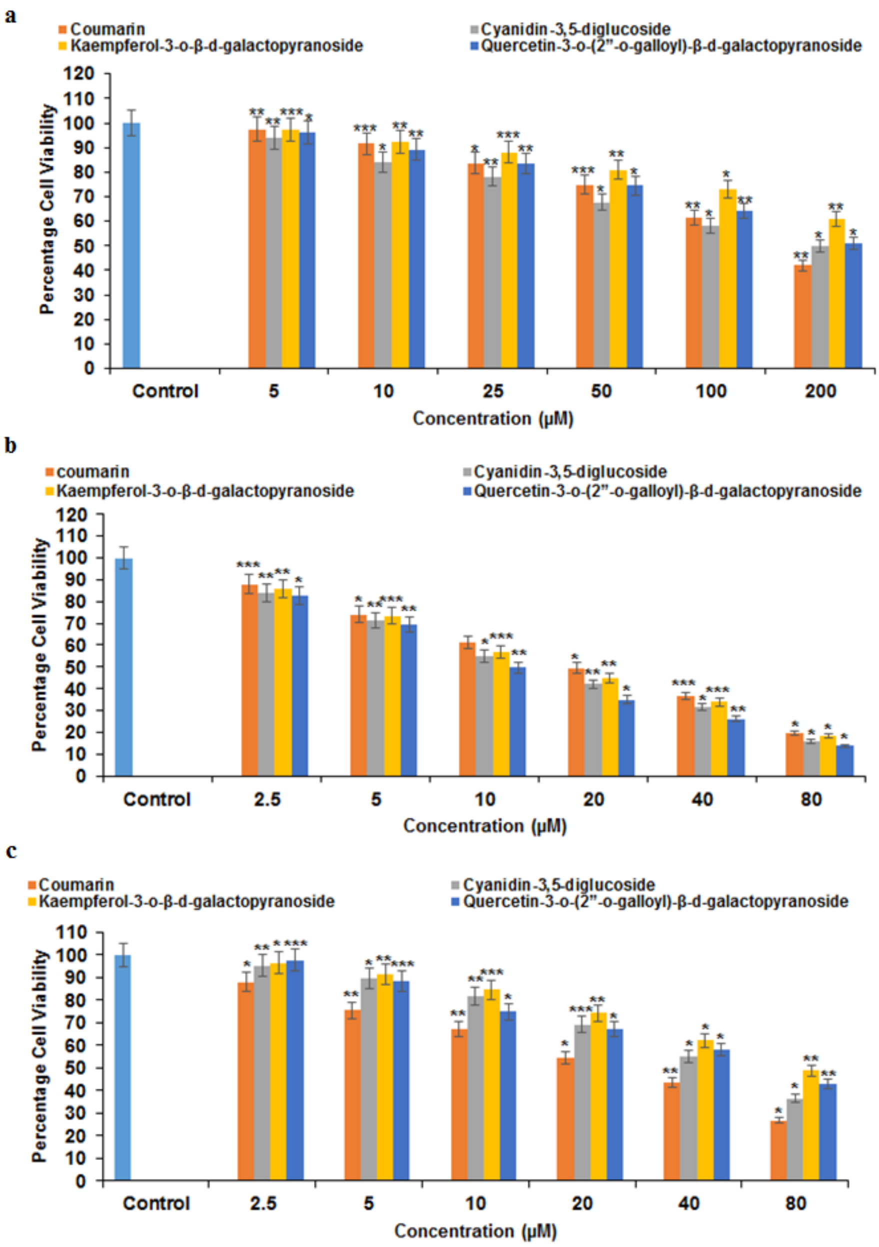

Supplementary Figure 1. Cytotoxic assay of (**a**). Compounds cyanidin-3,5-diglucoside, kaempferol-3-o-β-d-galactopyranoside and quercetin-3-o-(2”-o-galloyl)-β-d-galactopyranoside on HEK293 cells. (**b**) Anti-proliferation assay of compounds cyanidin-3, 5-diglucoside, kaempferol-3-o-β-d-galactopyranoside and quercetin-3-o-(2”-o-galloyl)-β-d-galactopyranoside on HeLa cells and (**c**). Compounds cyanidin-3, 5-diglucoside, kaempferol-3-o-β-d-galactopyranoside and quercetin-3-o-(2”-o-galloyl)-β-d-galactopyranoside on MCF-7 cells. Data represent three independent tests of experiments and results are shown as the mean ± SD. Coumarin is used as a positive control. *p < 0.05; **p < 0.001; ***p < 0.005 compared with the control.
